# A high-resolution large-scale dataset for building segmentation from aerial imagery in northeastern Italy

**DOI:** 10.1038/s41597-025-06014-4

**Published:** 2025-11-03

**Authors:** Claudio Rota, Flavio Piccoli, Rajesh Kumar, Gianluigi Ciocca

**Affiliations:** Department of Informatics, Systems and Communication, University of Milano-Bicocca, Milan, Italy

**Keywords:** Environmental sciences, Computer science

## Abstract

Accurate building segmentation from high-resolution aerial imagery is essential for numerous applications in remote sensing, urban planning, and disaster management. While AI-based methods enable fast, scalable, and cost-effective segmentation of building footprints, their development is often limited by the scarce availability of large-scale, geographically diverse datasets with reliable pixel-level annotations. In this work, we present SegFVG, a large-scale, high-resolution, and geographically diverse dataset for building segmentation, focused on the Friuli Venezia Giulia region in northeastern Italy. The dataset includes over 15,000 true orthophoto aerial image tiles, each of size 2000 × 2000 pixels with a ground sampling distance of 0.1 meters, paired with precise pixel-level building segmentation masks. Covering approximately 616 km^2^, SegFVG captures a broad spectrum of urban, suburban, and rural settings across varied landscapes, including mountainous, flat, and coastal areas. Alongside the dataset, we provide benchmark results using several deep learning models. These support the usability of SegFVG for the development of accurate segmentation models and serve as a baseline to accelerate future research in building segmentation.

## Background & Summary

Building segmentation refers to the task of identifying and delineating building footprints at the pixel level from aerial or satellite imagery. It plays a crucial role in a wide range of geospatial applications, including remote sensing, urban planning, environmental monitoring, infrastructure management, and disaster response^1–3^. In these domains, AI-based tools for automatic building segmentation have become increasingly essential, offering fast, scalable, and cost-effective solutions for extracting building footprints. The availability of these tools provides an opportunity to automate traditionally manual and resource-intensive mapping workflows^4^.

In recent years, deep learning has emerged as a highly effective approach for building segmentation, with models based on convolutional neural networks (CNNs) achieving remarkable performance. These models can learn hierarchical and contextual features directly from the input RGB imagery, allowing them to accurately delineate buildings across diverse environments and architectural styles^5^. The effectiveness of these models is strongly dependent on the availability of large-scale and high-quality annotated training datasets^6^. Such datasets must have high spatial resolution to accurately capture fine architectural details, and geographic diversity to ensure robustness across different urbanization levels and landscapes. Additionally, they must provide a sufficient volume of images to enable effective training of modern models and to ensure strong generalization performance.

Various datasets for building segmentation from aerial and satellite imagery have been proposed to support the training and evaluation of deep learning-based segmentation models. Table 1 shows a comparison among existing datasets.

**Table 1:** Comparison of existing datasets for building segmentation.

Dataset name	Images	Tile size (pixels)	Resolution (m)	Covered area (km^2^)	Building footprints	Urbanization level	Type	Geographical region
INRIA^8^	360	5,000	0.3	810	210,365	Urban	Aerial	Chicago, Kitsap County, Bellingham, San Francisco, Bloomington (the US) and Vienna, West Tyrol, Innsbruck, East Tyrol (Austria)
Massachusetts^13^	151	1500	1	340	N/A	Urban, suburban, and rural	Aerial	Massachusetts (the US)
ISPRS Potsdam^7^	38	6,000	0.05	4	N/A	Urban	Aerial	Potsdam (Germany)
WHU^29^	8,188	512	0.3	450	~ 220,000	N/A	Aerial	Christchurch (New Zealand)
LandCover.ai^9^	41	~ 4,000-9,000	0.25-0.5	216	N/A	Rural	Aerial	Poland
Spacenet 6^10^	20,408	900	0.5	120	~ 48,000	Urban, suburban, and rural	Satellite	Rotterdam (The Netherlands)
CBIS^12^	7,260	500	0.3	124	63,886	Urban and suburban	Satellite	Beijing, Shanghai, Shenzhen, Wuhan (China)
UBC^14^	800	600	0.5-0.8	66	~ 61,000	Urban	Satellite	Beijing (China) and Munich (Germany)
GF-7^11^	5,175	512	0.65	573	170,015	Urban, suburban, and rural	Satellite	Tianjin, Lanzhou, Chongqing, Ningbo, Guangzhou, Shenzhen (China)
SegFVG^15^	15,403	2,000	0.1	616	356,923	Urban, suburban, and rural	Aerial	Friuli Venezia Giulia region (Italy)

Although many datasets exist, none of them is simultaneously large-scale with a high spatial resolution and broad geographic diversity. For example, the ISPRS Potsdam dataset^7^ offers very high spatial resolution but is geographically limited, covering only a single urban area of Potsdam, in Germany. The INRIA dataset^8^ provides imagery from multiple locations around the world, but it is limited to urban areas and does not include rural or suburban regions, while the Landcover.ai dataset^9^ only focuses on rural areas across Poland. This geographic bias may limit the model generalization across diverse urbanization levels and building architectural styles. Datasets like Spacenet 6^10^, GF-7^11^, and CBIS^12^ provide broader geographic diversity, including many urban, suburban, and rural areas, but lack the spatial detail necessary for fine-grained segmentation. Similarly, the Massachusetts dataset^13^ offers a good geographic coverage and diversity, but its spatial resolution is relatively low (only 1 meter per pixel). This limited spatial detail may reduce the accuracy in capturing fine architectural features, such as small building footprints or complex roof structures. Other datasets, such as UBC^14^, are relatively small in scale (only 66 km^2^), limiting their usability for the training of modern deep learning models. Recently, the Microsoft Building Footprints dataset (https://planetarycomputer.microsoft.com/dataset/ms-buildings) has been proposed: it includes over 999 million buildings worldwide, obtained from Bing Maps imagery collected between 2014 and 2021. However, it may suffer from temporal misalignment between imagery and annotations, leading to inconsistent footprints. In addition, it is generated entirely through automated deep learning methods without validation from authoritative cartographic sources, which may affect its accuracy and reliability in certain regions. For all these reasons, there is the need for a large-scale, high-resolution, and geographically diverse dataset that provides accurate and reliable pixel-level building annotations.

**Fig. 1:**
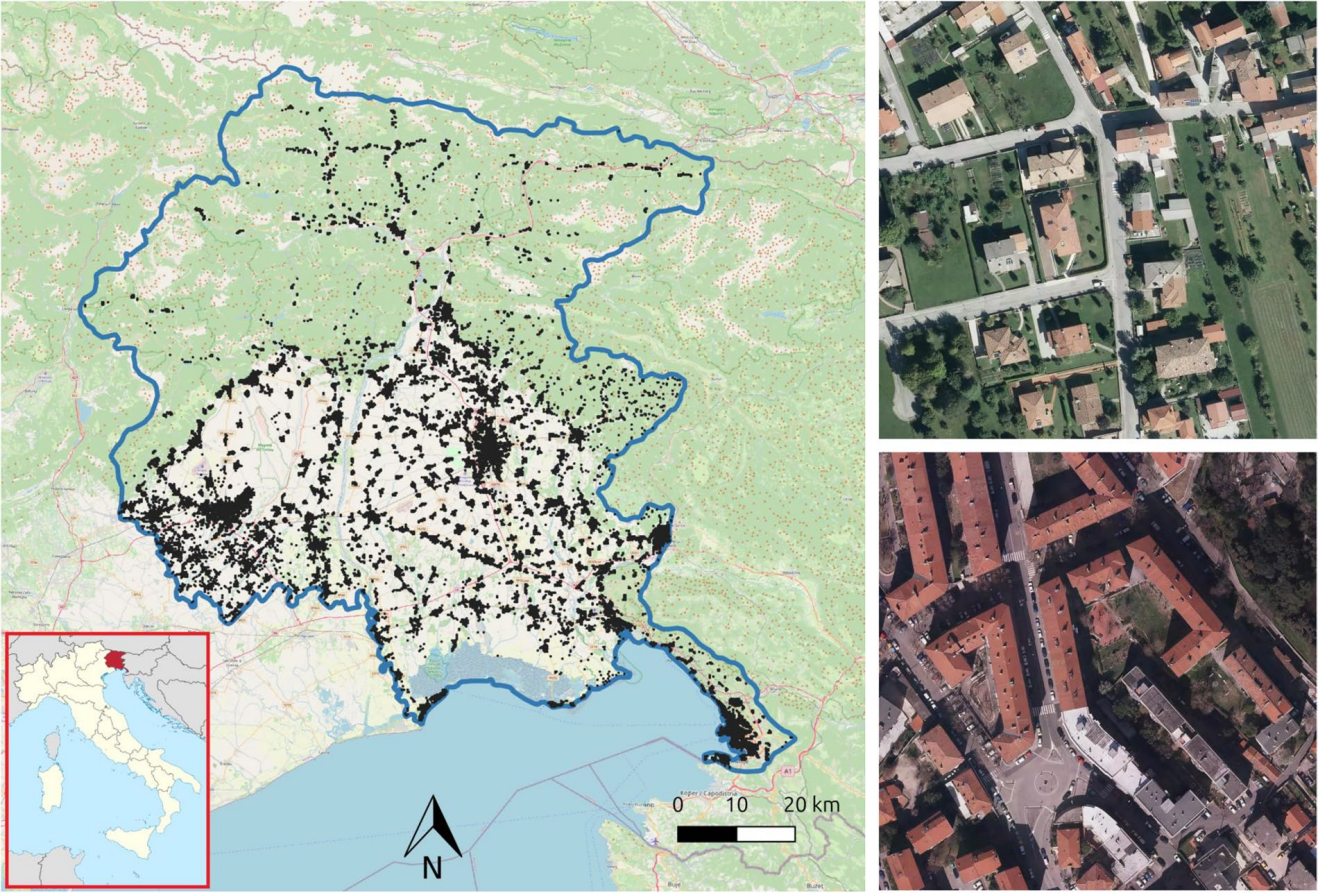
Spatial distribution of SegFVG image tiles (left) across the Friuli Venezia Giulia region. Each black dot represents an image tile of 2000 × 2000 pixels (200 × 200 m, examples on the right) included in the dataset, illustrating the geographic coverage.

**Fig. 2:**
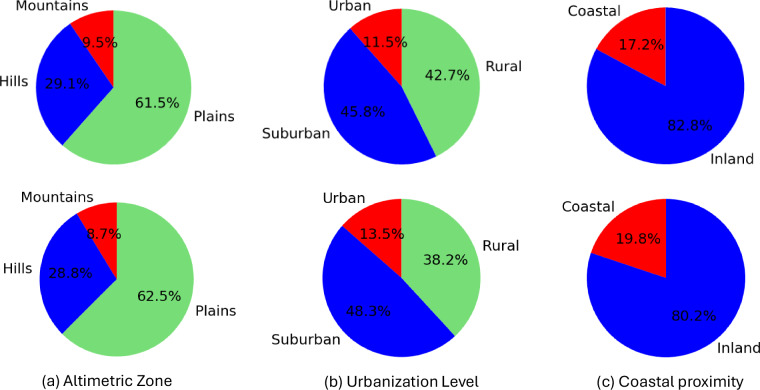
Overview of the dataset composition based on altimetric zone, urbanization level, and coastal proximity classification. In the top row, the percentages refer to the tiles in each class, while in the bottom row, they refer to the number of buildings in each class.

Given the limitations of existing datasets for building segmentation, in this work we introduce *Segmentation Friuli Venezia Giulia* (SegFVG)^15^, a large-scale, high-resolution dataset containing 15,403 aerial image tiles, each of size 2000 × 2000 pixels, with a ground sampling distance of 0.1 meters. SegFVG^15^ includes precise pixel-level annotations of building footprints across 616 km^2^ of the Friuli Venezia Giulia region in northeastern Italy. The area of Friuli Venezia Giulia is particularly interesting for building segmentation as it encompasses a diverse range of environments, including alpine rural zones in the north, flat agricultural plains in the center, and densely populated coastal settlements along the Adriatic Sea. The geographical distribution of the tiles contained in SegFVG^15^, as well as tile examples, is shown in Fig. 1. Each tile is represented by a black dot on the map. According to the classifications provided by the National Institute of Statistics (Istat) (https://www.istat.it/classificazione/principali-statistiche-geografiche-sui-comuni/), as shown in Fig. 2 (top row), SegFVG^15^ captures a diverse distribution of altimetric zones: 9.5% of the tiles are located in mountainous areas, 29.1% in hilly areas, and the remaining 61.5% in flat plains. In terms of urbanization levels, 11.5% of the tiles correspond to urban contexts, 45.8% to suburban areas, and 42.7% to rural settings. Finally, with respect to coastal proximity, 17.2% of the tiles are situated near the Adriatic Sea (coastal), while the remaining 82.8% are inland.

**Fig. 3:**
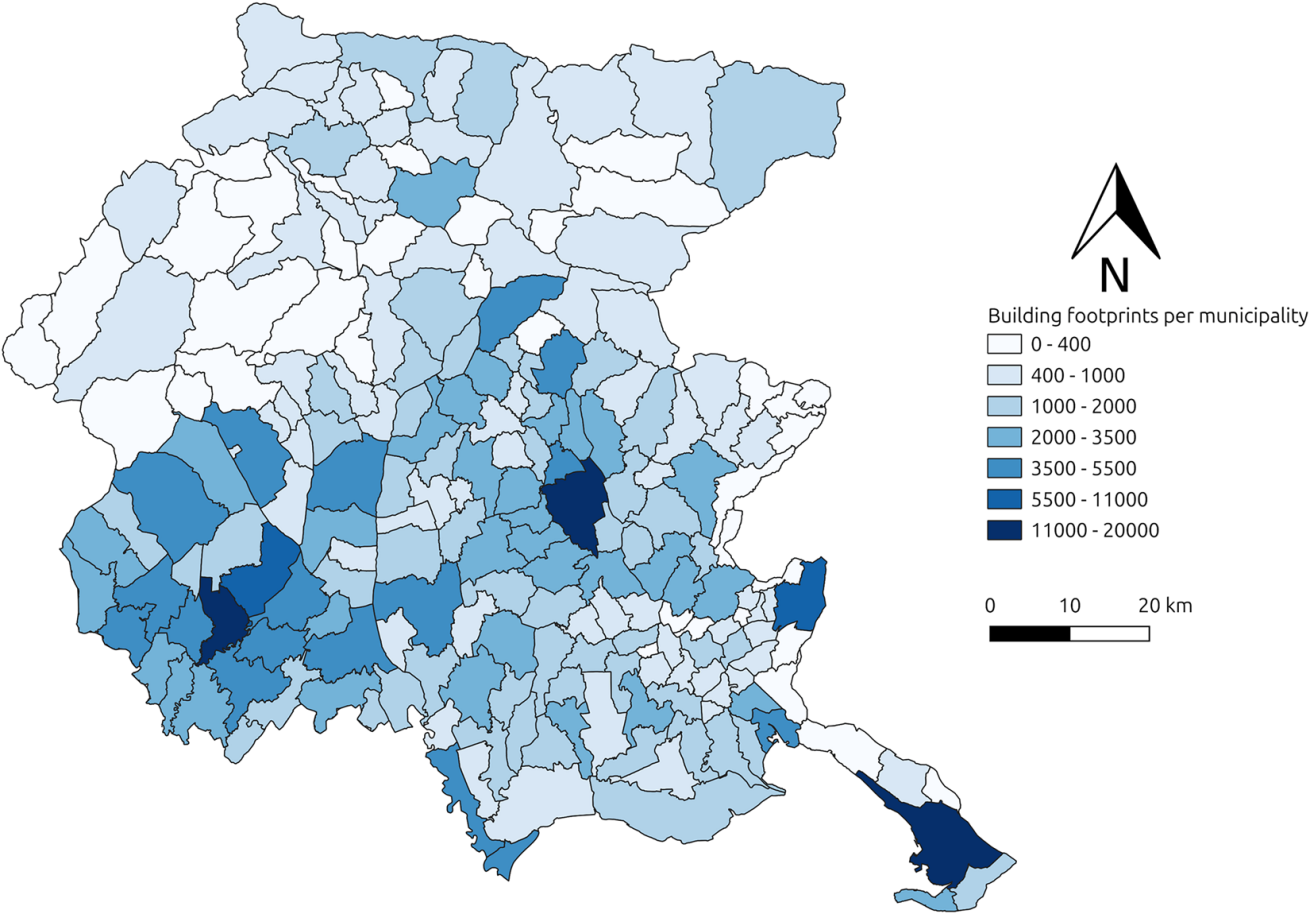
Map of the Friuli Venezia Giulia region showing the number of annotated buildings in the SegFVG dataset. There are a total of 215 municipalities. Each area refers to a municipality and is color-coded according to the total count of buildings, showing the concentration of annotations across the region.

In total, SegFVG^15^ includes approximately 357,000 annotated building structures. Figure 2 (bottom row) shows the building distribution according to their altimetric zone, urbanization level, and coastal proximity, while Fig. 3 illustrates the distribution of these buildings grouped by municipality, highlighting the spatial variability in building density across the region, from dense urban centers to sparsely populated rural areas. The combination of varied landscapes, urbanization levels, and architectural styles creates a representative and challenging benchmark for analyzing buildings across different geographic and urban contexts.

Overall, SegFVG^15^ is characterized by its *large scale*, its *high spatial resolution*, and its *geographic diversity* (i.e., it includes heterogeneous environments, such as coastal areas, plains, hills, and Alpine regions, which are associated with distinct settlement patterns and building typologies). These features make it particularly well-suited for the development of deep learning models for building segmentation.

In addition to the dataset, we provide benchmark results using multiple deep learning models, which demonstrate the usability of SegFVG^15^ for the development of accurate segmentation models and can be used as a baseline for future research in this field. To the best of our knowledge, SegFVG^15^ is the first publicly available building segmentation dataset focused on the Italian territory.

## Methods

**Fig. 4:**
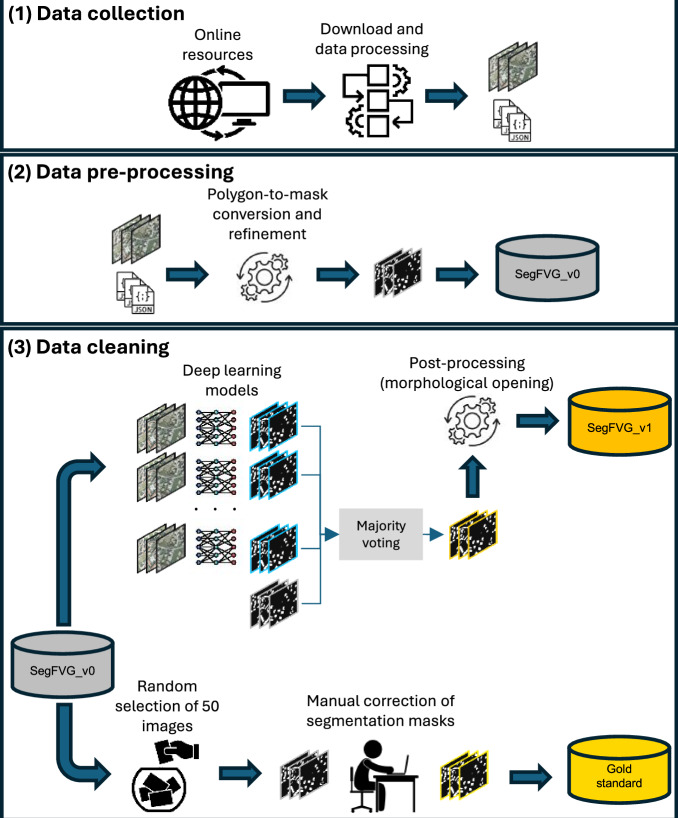
The framework used for the generation of the SegFVG dataset.

An overview of the framework we adopted to create SegFVG^15^ is shown in Fig. 4. It involves three main steps: (1) data collection, (2) data processing, and (3) data cleaning. In the following, we describe these steps more in detail.

### Data collection

We constructed the SegFVG^15^ dataset by leveraging official geospatial services provided by the Friuli Venezia Giulia (FVG) region. The data collection process begins with retrieving building shapes from the regional Web Feature Service (WFS) (https://serviziogc.regione.fvg.it/geoserver/EDIFICI/wfs). These layers provide building volumetric units for the entire region derived from the Regional Technical Numerical Map (RTNM) (https://eaglefvg.regione.fvg.it/). A volumetric unit is defined as the smallest portion of a building that has a uniform elevation from the ground (https://geoportale.comune.milano.it/sit/tematiche/territorio/).

We first reprojected all vector layers to the EPSG:6708 coordinate reference system. We divided the FVG region into square tiles measuring 200 meters per side to organize the spatial data. We then discarded tiles containing fewer than 40 shapes (volumetric units) to prioritize areas with meaningful building density and to reduce uninformative background regions.

For each retained tile, we downloaded true orthophotos with a ground sampling distance of 0.1 meters per pixel from the regional Web Map Service (WMS) (http://irdat-ortofoto.regione.fvg.it/geoserver/ortofoto/ows). These images were captured during aerial surveys conducted between 2017 and 2020 using aircraft equipped with Vexcel UltraCam Eagle and UltraCam Xp digital large-format cameras. We adopted a sliding window approach to manage the retrieval of images spanning large shapes. All resulting images are saved as GeoTIFFs to preserve accurate georeferencing metadata.

We clipped shapes to match tile boundaries and filtered them by a minimum area threshold of 20 m^2^ to remove small and potentially noisy shapes. We converted each remaining shape into a polygon defined in image pixel coordinates using an affine transformation and applied a y-axis inversion to align with the image coordinate system. Finally, the polygon boundaries are serialized into JSON format.

For each tile, we generated a pair of files: a raster RGB image and a corresponding JSON annotation file containing the filtered volumetric units of each building represented in the image.

### Data pre-processing

Following the data collection phase, the raw dataset consisted of raster RGB images paired with volumetric unit information provided as JSON files. As a first step, we discarded a subset of images that exhibited large black regions in the background. These areas, represented by zero-valued pixels, are likely the result of incomplete image tiles, which occur when the tile contains areas that fall outside the boundaries of the Friuli Venezia Giulia region. We then converted the vector polygons in the JSON files into rasterized binary masks, assigning a value of 1 to pixels within the polygons and 0 to all other pixels. During this process, all connected volumetric units are aggregated to produce a single unified footprint for each building. This step is essential because, in the RGB true orthoimages, these structures appear as continuous buildings without internal divisions, and treating each volumetric unit separately creates artificial splits that do not reflect the visual reality. The dataset obtained after these pre-processing operations consists of image/mask pairs. We refer to this preliminary dataset version as *SegFVG_v0*.

### Data cleaning

After manually inspecting a random subset of images from SegFVG_v0, we identified recurring annotation errors, such as missing buildings (false negatives) and incorrectly labeled structures (false positives). These issues are likely due to temporal discrepancies between the imagery and the RTNM data, as they were captured at different times, leading to mismatches caused by construction, demolition, or changes in land use. An example is presented in Fig. 7a, which shows both missing building footprints and erroneous annotations.

These inaccuracies can negatively impact both the training of deep learning models and the reliability of performance evaluations^16^. We thus implemented a data cleaning pipeline to improve the overall annotation quality, and we established a manually corrected reference set to quantitatively assess the effectiveness of the data cleaning procedure.

We first randomly selected 50 images from the SegFVG_v0 dataset and manually refined their corresponding masks using the CVAT tool (https://www.cvat.ai/). The goal was to ensure that all building footprints were accurately annotated, removing both false positives and false negatives. This manually corrected subset, referred to as the *gold standard*, is used as a reference for evaluating the effectiveness of the data cleaning procedure.

We then implemented a semi-automatic data cleaning procedure to address the annotation inconsistencies. In short, we trained different deep learning models for building segmentation with different backbones using all the images in the SegFVG_v0 dataset, excluding the 50 images in the gold standard, and finally used the majority voting consensus of their predictions to generate the corrected segmentation masks. The use of multiple backbones allows leveraging the complementary strengths of different feature extractors, which can capture different semantic patterns from the training data, even if they contain some inconsistencies^17^. Specifically, we used four models based on the U-Net architecture^18^ with Resnet-50^19^, Efficientnet-B4^20^, Densenet-201^21^, and Xception^22^ as backbone encoders. Each encoder is initialized with pre-trained weights from ImageNet^23^. During training, the batch size is set to 32 and the learning rate to 1e-4. We use the Dice loss ${{\mathcal{L}}}_{Dice}$, which is defined as:

1 $${{\mathcal{L}}}_{Dice}=1-2\cdot \frac{\sum {y}_{pred}\cdot {y}_{true}}{\sum {y}_{pred}+\sum {y}_{true}+\epsilon },$$

where *y*_*pred*_ ∈ [0, 1] is the predicted probability after sigmoid activation, *y*_*true*_ ∈ {0, 1} is the reference label, and *ϵ* is a small value to ensure numerical stability. We trained the models for 12,000 iterations. For data augmentation, we randomly cropped images at 256 × 256 pixel resolution and used various strategies, including flip, rotation, and corrections applied to brightness, contrast, hue, and saturation.

After all the models were trained, we used each of them to generate segmentation masks for the entire dataset. In addition to these four predictions, we considered the masks of SegFVG_v0, resulting in five candidate masks per image. We then applied majority voting consensus across these five candidates to obtain a refined segmentation mask, where each pixel was labeled as building (i.e., 1) if at least three of the five masks agreed. Formally, let *M* = {*m*_1_, *m*_2_, *m*_3_, *m*_4_, *m*_5_} be the set of binary masks, where each *m*_*i*_ ∈ {0, 1}^*H*×*W*^ is a binary mask of height *H* and width *W*, *m*_*i*_(*x*, *y*) denotes the pixel value at location (x,y) in the i-th mask, with 1 indicating a building pixel and 0 indicating the background. Then, $\widehat{m}\in {\{0,1\}}^{H\times W}$ is the refined mask and $\widehat{m}(x,y)$ is defined as:

We finally post-processed the obtained mask by applying morphological opening (erosion followed by dilation) using a 13 × 13 kernel, which helped remove small spurious regions.

The dataset obtained after these data cleaning operations is called *SegFVG_v1*, which corresponds to the final version.

## Data Record

**Fig. 5:**
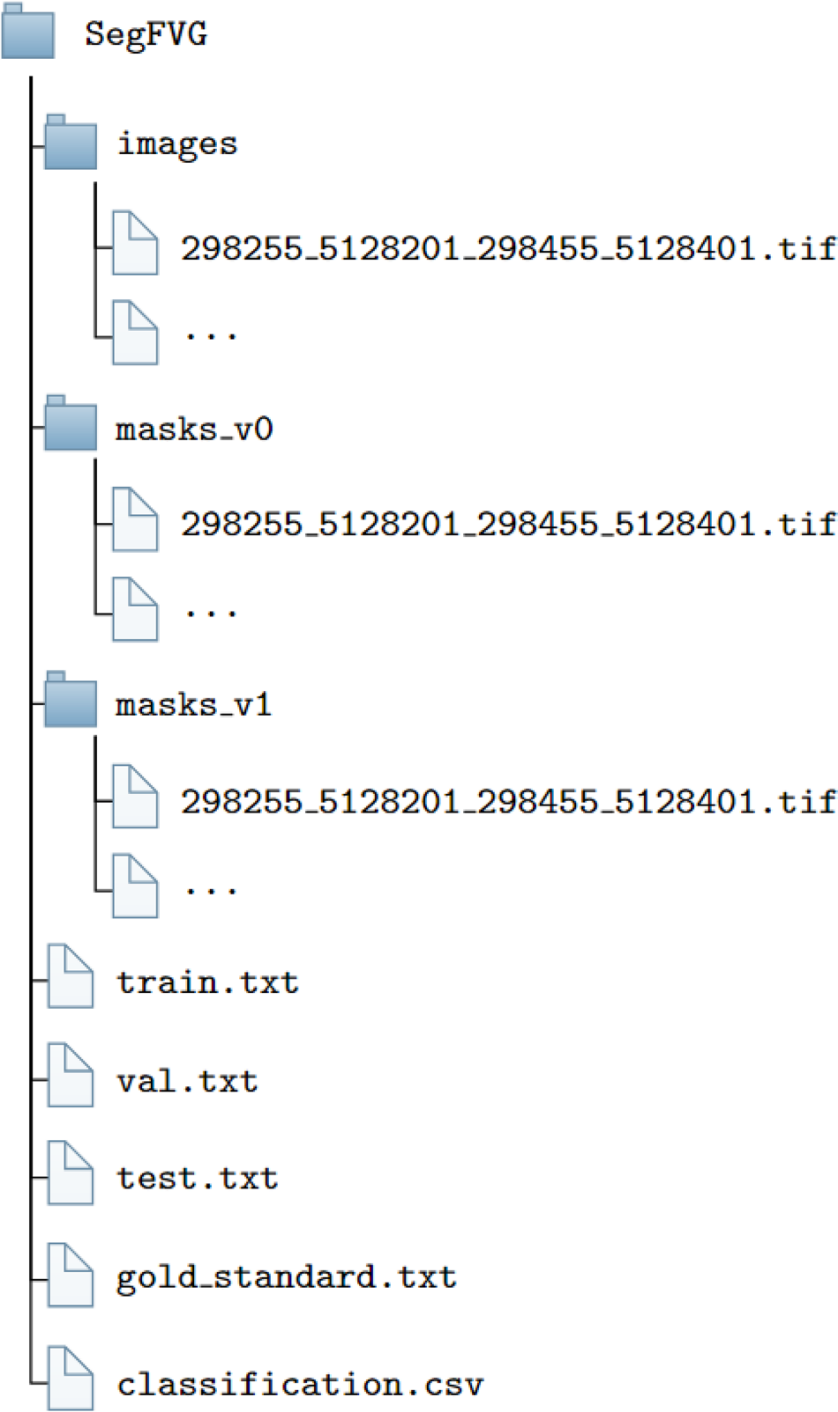
Directory structure of the SegFVG dataset.

The SegFVG^15^ dataset is available for download at 10.17632/9kbc6zdn7b. It is distributed as a compressed ZIP archive of approximately 24 GB. The dataset presented and peer reviewed in this article corresponds to *Version 2*. Figure 5 illustrates the hierarchical directory structure.

**Fig. 6:**
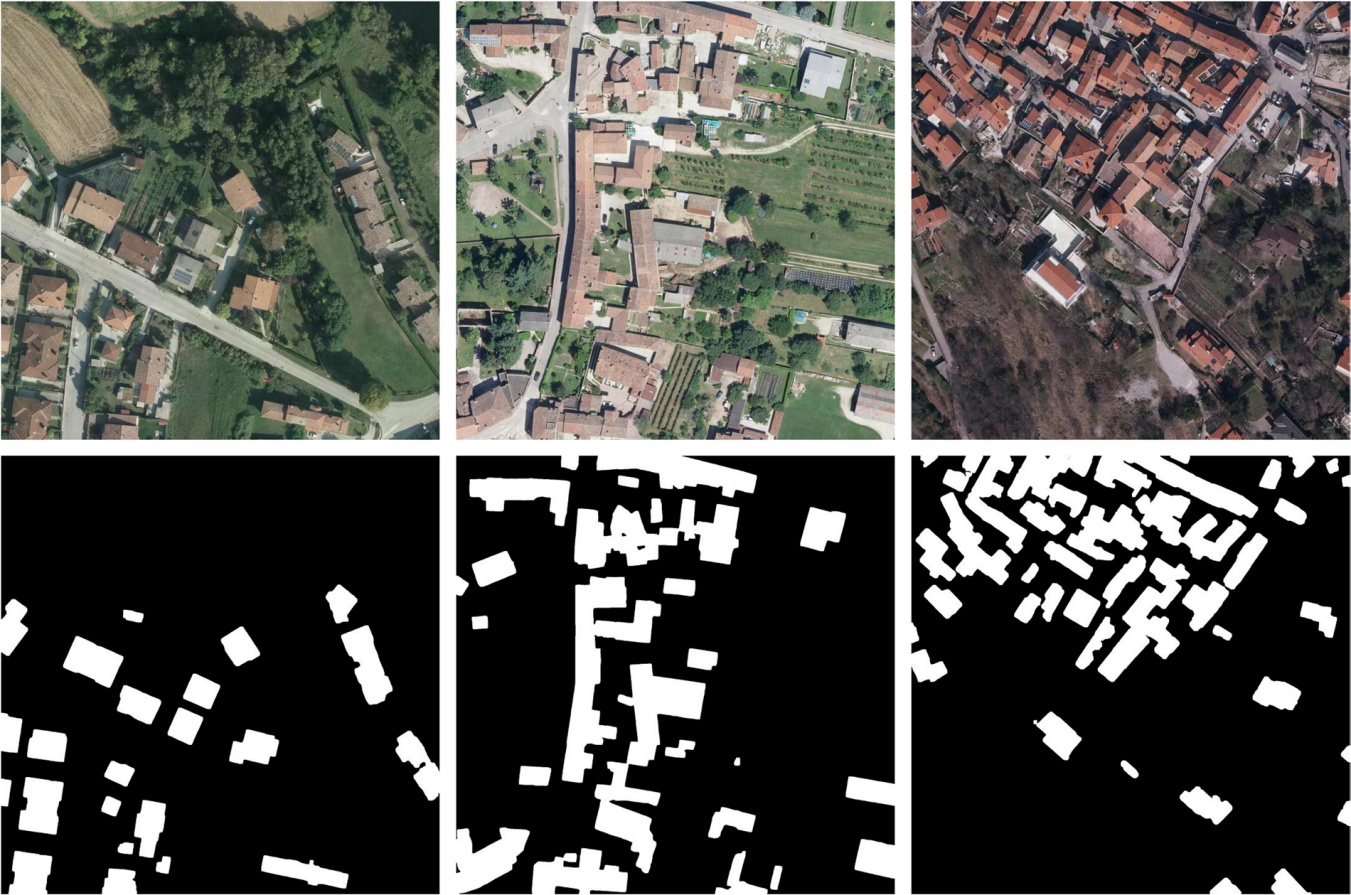
Examples of aerial image tiles (top) and their corresponding building segmentation mask (bottom). The masks highlight building footprints in white.

The root folder, named *SegFVG*, contains three subdirectories: *images*, *masks_v0*, and *masks_v1*. They store the input RGB aerial images and their corresponding segmentation masks, where masks_v0 contains the preliminary annotations (SegFVG_v0), and masks_v1 contains the cleaned annotations produced by the data cleaning pipeline (SegFVG_v1). Each RGB image is associated with a segmentation mask that provides precise pixel-level annotations of building footprints. Some examples are shown in Fig. 6.

All files are stored in GeoTIFF format and named consistently so that each image corresponds directly to its reference mask. Each image filename encodes the spatial extent of the corresponding tile using projected coordinate values. Specifically, the filename follows the structure ${{\rm{x}}}_{\min }\_{{\rm{y}}}_{\min }\_{{\rm{x}}}_{\max }\_{{\rm{y}}}_{\max }.{\rm{tif}}$, where each value represents the coordinates of the tile boundary box (upper left and bottom right corners) in the projected coordinate reference system EPSG:6708.

The dataset is split into training, validation, and test partitions, which are provided as TXT files in the root directory: *train.txt*, *val.txt*, and *test.txt*. Each TXT file lists the filenames corresponding to the images in that split. Specifically, the training, validation, and test sets contain 10,748, 1,535, and 3,070 images, respectively. In addition, the root directory contains a *gold_standard.txt* file, which includes the filenames of the 50 manually corrected images and segmentation masks used to evaluate the data cleaning procedure. The corresponding masks before and after manual correction are placed in masks_v0 and masks_v1, respectively. The description of the dataset splits is reported in Table 2.

**Table 2:** Overview of the SegFVG splits, including the number of image tiles, total buildings annotated, and total geographic area covered (in km^2^) for each split.

Split name	Num of Tiles	Num. of buildings	Covered area (km^2^)
Train	10,748	249,666	430
Validation	1,535	35,301	61
Test	3,070	70,531	123
Gold standard	50	1,425	2
Total	15,403	356,923	616

Finally, following the classifications described in Section “Background & Summary”, we provide a CSV file named *classification.csv*, which contains information for each image tile, including the municipality it belongs, its associated altimetric zone (mountain, hill, or plain), urbanization level (urban, suburban, or rural), and coastal proximity (coastal or inland). Table 3 shows some examples of records in the classification.csv file.

**Table 3:** Examples of the *classification.csv* file content.

Tile	Municipality	Altimetric zone	Urbanization level	Coastal proximity
403223_5050937_403423_5051137	Muggia	hill	suburban	coastal
302255_5129201_302455_5129401	Cimolais	mountain	rural	inland
302783_5091192_302983_5091392	Sacile	plain	suburban	inland
397623_5064937_397823_5065137	Trieste	hill	urban	coastal

Each tile is associated with information about the municipality, altimetric zone, urbanization level, and coastal proximity.

## Technical Validation

We validated the quality of the segmentation masks obtained using the data cleaning pipeline described in Section “Data cleaning”. In addition, we conducted a series of experiments using standard deep learning models to assess the quality and usability of SegFVG^15^ for building segmentation from aerial imagery. The results are evaluated using standard metrics for semantic segmentation, i.e., pixel-level Precision, Recall, F1-score, and Intersection over Union (IoU).

### Quality assessment of data cleaning pipeline

With this evaluation, we aim to validate the data cleaning pipeline we used to correct the errors we identified in SegFVG_v0, as described in Section “Data cleaning”. Indeed, the annotations in SegFVG_v0 may contain errors that typically appear as false negatives, where buildings are missed, or false positives, where background regions are incorrectly labeled as buildings.

**Fig. 7:**
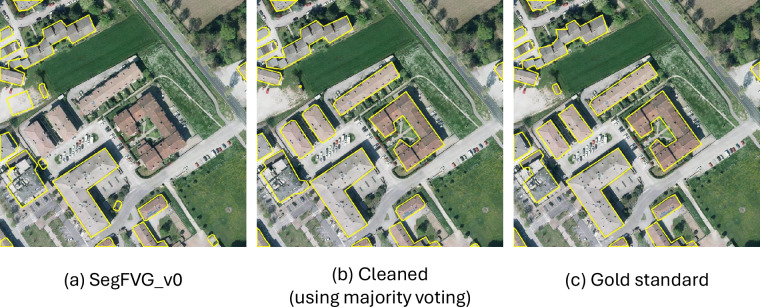
Example of the images obtained using the data cleaning process.

We validated the quality of the data cleaning pipeline using the gold standard. The performance of the models involved in this process is reported in Table 4. As shown, the direct comparison of the segmentation masks in SegFVG_v0 with the gold standard reports an F1-score and IoU of 0.856 and 0.749, respectively. This highlights the presence of annotation errors and motivates the need to clean the data. We can consider these results as the baseline. The performance obtained by the different deep learning models shows an improvement over the baseline, confirming that model-based refinement is an effective solution to identify and correct annotation errors. Finally, when the model predictions are combined and integrated with the annotations of SegFVG_v0, the resulting masks achieve a higher agreement with the gold standard. Specifically, the performance improves to 0.947 and 0.900 in F1-score and IoU, respectively, demonstrating a considerable gain in segmentation quality. Since the gold standard contains manually corrected and error-free annotations, higher performance on this set suggests that the cleaned annotations are more accurate and of better quality. This is also visually confirmed by Fig. 7, which shows an example of segmentation masks from SegFVG_v0 cleaned using the adopted pipeline. Here we polygonized the segmentation masks and displayed only the polygon boundaries for better clarity. We can see that the cleaned masks are more similar to the gold standard.

**Table 4:** Performance of different data cleaning strategies evaluated on the gold standard.

Data cleaning strategy	Precision	Recall	F1-score	IoU
—	0.895	0.821	0.856	0.749
Resnet-50	0.943	0.913	0.928	0.865
Efficientnet-B4	0.842	0.912	0.927	0.863
Densenet-201	0.908	0.945	0.926	0.863
Xception	0.929	0.941	0.935	0.879
Majority voting	0.949	0.946	0.947	0.900

The first row corresponds to the SegFVG_v0 annotations (i.e., no data cleaning).

These results validate both numerically and visually the reliability of the proposed data cleaning pipeline, which significantly improved the quality of the annotations compared to those of SegFVG_v0.

### Performance of deep learning models

The goal of this evaluation is to assess whether the SegFVG^15^ can support the training of deep learning models for building segmentation from aerial imagery.

We selected different architectures, including U-Net^18^, which is a well-established encoder-decoder model; Pyramid Scene Parsing Network (PSPNet)^24^, which aggregates context using pyramid pooling at different spatial resolutions; Feature Pyramid Network (FPN)^25^, which enhances semantic segmentation by combining high-level semantic features with low-level spatial details across scales; DeepLabV3^26^, which leverages atrous spatial pyramid pooling to capture multi-scale contextual information; Pyramid Attention Network (PAN)^27^, which integrates multi-scale contextual information through spatial attention mechanisms; and Segmentation Transformer (SegFormer)^28^, which is a transformer-based model known for its efficiency and accuracy. We followed the same training setup adopted for the models used for the data cleaning pipeline, as described in Section “Data cleaning”. The only difference is the number of training iterations, which is here set to 8,400. The hyperparameters we used for these experiments are summarized in Table 5.

**Table 5:** Summary of the hyperparameters used during the training of deep learning models.

Hyperparameter	Value
Batch size	32
Learning rate	1e-4
Optimizer	Adam
Initial weights	ImageNet
Iterations	8400
Patch size	256 × 256
Loss function	Dice
Geometric augmentation	Flip, rotation
Color augmentation	Brightness, contrast, hue, saturation

We report the experimental results obtained on the test set, using SegFVG_v1 masks as a reference, in Table 6. These results can be used as a baseline for future research. In addition to evaluating model performance, the table reports the total number of model parameters, the training time required to complete 8400 iterations using the previously discussed setup, and the inference time required to generate a segmentation map from an input image of size 2000 × 2000. Time is measured using a NVIDIA GeForce GTX 1080 GPU.

**Table 6:** Comparative evaluation of different deep learning models for building segmentation on the test set.

Model	Backbone	Precision	Recall	F1-score	IoU	Param.	Train. time	Infer. time
UNet	Resnet-101	0.943	0.975	0.959	0.921	51M	1h 27m	417 ms
PSPNet	Resnet-101	0.885	0.759	0.817	0.691	43M	7h 50m	142 ms
FPN	Resnet-101	0.934	0.962	0.948	0.901	45M	1h 13m	348 ms
DeepLabV3	Resnet-101	0.934	0.956	0.945	0.896	46M	2h 48m	427 ms
PAN	Resnet-101	0.860	0.965	0.910	0.834	43M	1h 34m	384 ms
SegFormer	MiT-B3	0.955	0.940	0.947	0.900	45M	2h 22m	943 ms

The training time is the time required to complete model training. The inference time is the time required to process an image of 2000 × 2000 pixel resolution.

**Fig. 8:**
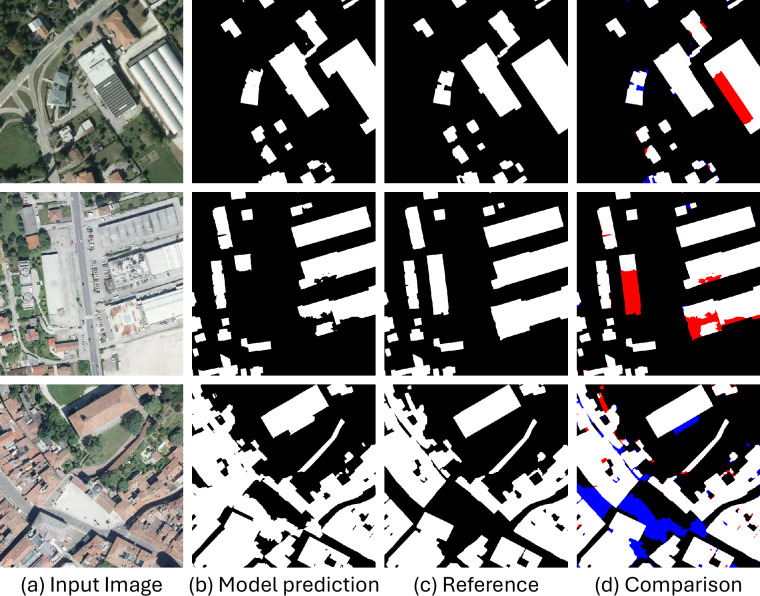
Examples of building segmentation masks obtained using UNet^18^. In Fig. 8d, white pixels are true positives, red ones are false negatives, blue ones are false positives.

Looking at precision, SegFormer^28^ achieves the highest value (0.955), showing its effectiveness in minimizing false positives. In terms of recall, UNet^18^ achieves the highest value (0.975), indicating a stronger ability to detect most pixels corresponding to buildings. Among all models, UNet^18^ also achieves the highest F1-score of 0.959, indicating a strong balance between precision and recall. In contrast, PSPNet exhibits the lowest performance with an F1-score of 0.817. Regarding IoU, UNet^18^ again performs best with a score of 0.921, confirming its robustness across different metrics. Overall, UNet is the best-performing model. In contrast, PSPNet^24^ is the worst-performing model, achieving F1-score and IoU values that are 0.142 and 0.230 lower, respectively, compared to UNet^18^. Fig. 8 shows some examples of building segmentation masks obtained using the UNet^18^ model. Specifically, Fig. 8d highlights the differences between the predicted masks and the reference ones, illustrating common errors made by building segmentation models. These include: (1) missed buildings (false negatives), where small or shadowed structures are not detected; (2) spurious detections (false positives), where non-building elements are incorrectly classified as buildings; and (3) boundary inaccuracies, where mask borders are overly smooth or slightly misaligned.

Using the UNet^18^ model, we conducted a more detailed performance analysis aiming to understand how segmentation performance varies across different geographical and demographic conditions present in the dataset. For reference, we also documented the performance of the other models.

As a first step, we evaluated model performance in regions with varying urbanization levels. Each image tile was categorized as urban, suburban, or rural according to the number of inhabitants per square kilometer. These categories often reflect different patterns in the distribution and structure of the building. Urban areas are typically characterized by compact layouts, where buildings are closely spaced and exhibit a wide variety of shapes and sizes.

In contrast, rural areas contain only a few isolated structures, often dispersed across large expanses of natural or agricultural land. The obtained results are summarized in Table 7.

**Table 7:** Performance of the models for building segmentation across areas categorized by population density, i.e., densely, moderately, and sparsely populated regions within the test set.

Urbanization level	Num. of tiles	Model	Precision	Recall	F1-score	IoU
Urban	363	UNet	0.931	0.967	0.949	0.902
		PSPNet	0.888	0.713	0.790	0.654
		FPN	0.928	0.942	0.935	0.878
		DeepLabV3	0.938	0.929	0.933	0.875
		PAN	0.816	0.966	0.885	0.793
		SegFormer	0.955	0.908	0.931	0.871
Suburban	1,396	UNet	0.944	0.978	0.960	0.924
		PSPNet	0.886	0.780	0.829	0.708
		FPN	0.934	0.966	0.950	0.905
		DeepLabV3	0.935	0.961	0.948	0.901
		PAN	0.863	0.967	0.912	0.839
		SegFormer	0.956	0.943	0.950	0.904
Rural	1,311	UNet	0.947	0.976	0.961	0.925
		PSPNet	0.883	0.753	0.813	0.685
		FPN	0.935	0.966	0.950	0.905
		DeepLabV3	0.931	0.962	0.946	0.898
		PAN	0.877	0.963	0.918	0.849
		SegFormer	0.953	0.951	0.952	0.908

**Fig. 9:**
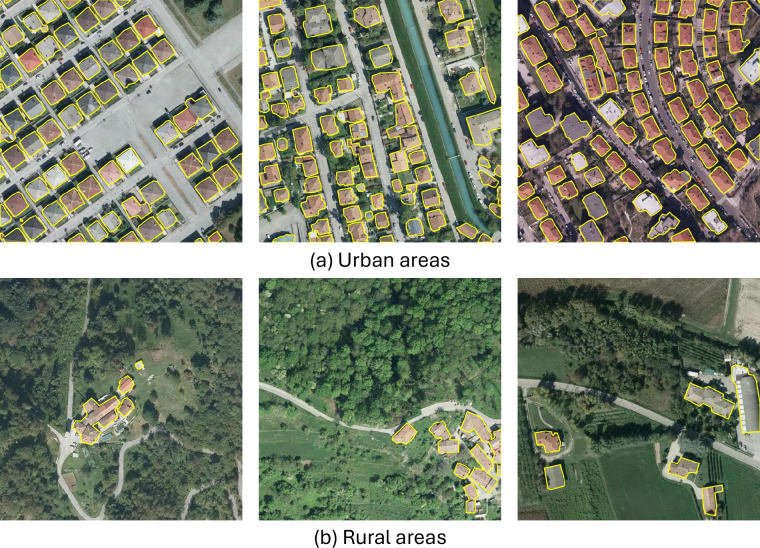
Example of UNet segmentation results on urban and rural areas.

The model demonstrates consistent performance across all urbanization levels, with F1-scores above 0.940 and IoU values above 0.900. Slightly lower performance in urban areas suggests challenges related to building overlap and structural complexity. In contrast, suburban and rural areas yield slightly better results, likely due to simpler spatial arrangements and clearer separation between buildings. Figure 9 shows some examples of segmentation results obtained in urban and rural areas.

We further analyzed the performance of the model in different types of landscapes by grouping image tiles into three categories according to their altimetric zone: mountains, hills, and plains. This geographic diversity often presents buildings with different characteristics: mountainous regions often contain scattered buildings reflecting the lower population density, hilly areas tend to feature moderately dense and varied structures, while plains typically exhibit more regular, densely arranged buildings. The results presented in Table 8 show that the model performs well in all types of landscapes.

**Table 8:** Performance of the models for building segmentation in different altimetric zones, i.e., mountains, hills, and plains within the test set.

Altimetric zone	Num. of tiles	Model	Precision	Recall	F1-score	IoU
Mountains	306	UNet	0.928	0.971	0.949	0.903
		PSPNet	0.863	0.602	0.709	0.549
		FPN	0.924	0.947	0.935	0.878
		DeepLabV3	0.904	0.958	0.930	0.869
		PAN	0.858	0.957	0.904	0.825
		SegFormer	0.939	0.950	0.944	0.895
Hills	885	UNet	0.943	0.973	0.958	0.919
		PSPNet	0.885	0.757	0.816	0.689
		FPN	0.939	0.955	0.947	0.899
		DeepLabV3	0.936	0.948	0.942	0.890
		PAN	0.846	0.966	0.902	0.821
		SegFormer	0.956	0.936	0.946	0.897
Plains	1,879	UNet	0.945	0.977	0.960	0.924
		PSPNet	0.887	0.778	0.829	0.708
		FPN	0.932	0.967	0.949	0.904
		DeepLabV3	0.937	0.960	0.948	0.901
		PAN	0.867	0.966	0.914	0.842
		SegFormer	0.956	0.941	0.948	0.902

**Fig. 10:**
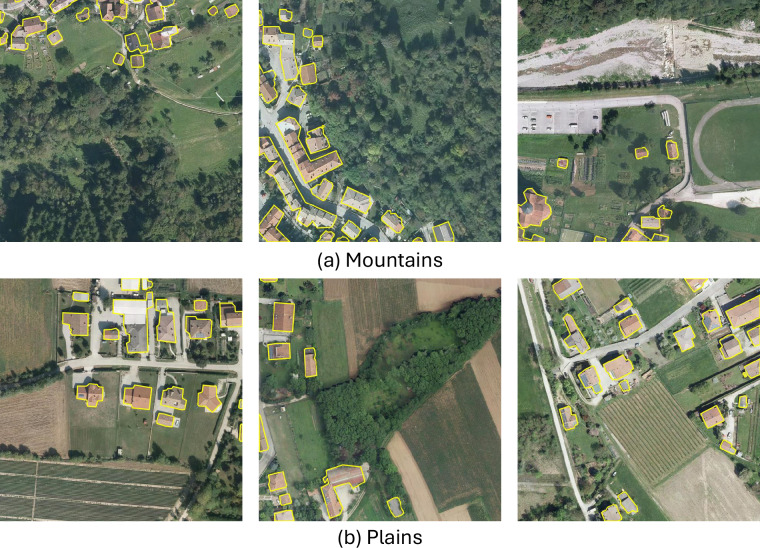
Example of UNet segmentation results on mountains and plains.

The lowest performance is observed in mountainous areas, where the model achieves an F1-score of 0.949 and an IoU of 0.903. This is likely due to visual and topographic complexity in such regions, including steep slopes, shadows, and more irregular building patterns. In contrast, performance improves slightly in hilly regions, with an F1-score of 0.958 and an IoU of 0.919, and reaches its highest levels in plains, where the model achieves an F1-score of 0.960 and an IoU of 0.924. The more uniform terrain and regular building layouts in plain regions likely contribute to this improved performance. Figure 10 shows examples of segmentation results obtained in mountainous and plain areas.

Finally, we investigated the model performance by separating coastal from inland areas. The results in Table 9 show that the model performs well in both contexts, but with slightly better results in inland areas. Specifically, the model achieves an F1-score of 0.961 and an IoU of 0.925 for inland areas, compared to an F1-score of 0.949 and an IoU of 0.903 in coastal areas. The lower performance in coastal zones may be attributed to the greater variability in building styles and densities, as well as visual interference from the sea, beaches, and other shoreline features.

**Table 9:** Performance of the models for building segmentation in coastal and inland areas within the test set.

Coastal proximity	Num. of tiles	Model	Precision	Recall	F1-score	IoU
Coastal	501	UNet	0.933	0.966	0.949	0.903
		PSPNet	0.892	0.685	0.775	0.633
		FPN	0.936	0.941	0.938	0.884
		DeepLabV3	0.932	0.933	0.932	0.873
		PAN	0.817	0.963	0.884	0.792
		SegFormer	0.953	0.917	0.934	0.877
Inland	2,569	UNet	0.945	0.977	0.961	0.925
		PSPNet	0.883	0.776	0.826	0.704
		FPN	0.933	0.967	0.950	0.904
		DeepLabV3	0.934	0.961	0.948	0.901
		PAN	0.871	0.966	0.916	0.845
		SegFormer	0.955	0.946	0.950	0.905

These results demonstrate the capability of SegFVG^15^ to support high-performance building segmentation from aerial imagery across diverse geographic and demographic contexts. The consistent performance observed in varying population densities and geographical contexts validates the representativeness of the dataset and its utility for downstream applications and model development.

## Data Availability

SegFVG^15^ is publicly available at 10.17632/9kbc6zdn7b under a CC BY 4.0 license, which permits reuse and modification with appropriate attribution. The dataset is distributed as the archive file *SegFVG.zip*

## Code availability

The source code for training deep learning models and performing inference, together with pretrained model checkpoints, is available in the archive *Code.zip* at 10.17632/9kbc6zdn7b. The code and checkpoints are released under a CC BY 4.0 license, permitting reuse and modification with appropriate attribution.
